# Effects of Acidic Exposure on Surface Properties and Bacterial Adhesion of Three Resin-Based CAD/CAM Materials

**DOI:** 10.3390/polym18151818

**Published:** 2026-07-25

**Authors:** Sarah Almuhayya, Rua Babaier, Eija Säilynoja, Ali M. Somily

**Affiliations:** 1 Department of Clinical Laboratory Science, College of Applied Medical Sciences, King Saud University, Riyadh 12372, Saudi Arabia; 2 Department of Prosthetic Dental Sciences, College of Dentistry, King Saud University, Riyadh 11545, Saudi Arabia; 3 Department of Biomaterials Science and Turku Clinical Biomaterial Center—TCBC, Institute of Dentistry, University of Turku, 20014 Turku, Finland; eija.sailynoja@gc.dental; 4 Research Development and Production Department, Stick Tech Ltd.—A GC Europa AG Company, 20520 Turku, Finland; 5 Department of Pathology and Laboratory Medicine, College of Medicine, King Saud University, King Saud University Medical City, Riyadh 11451, Saudi Arabia; somily@ksu.edu.sa

**Keywords:** CAD/CAM, resin composites, *Streptococcus mutans*, biofilm adhesion, wettability, surface roughness, confocal laser scanning microscopy, scanning electron microscopy

## Abstract

CAD/CAM reinforced resin composites are increasingly used in restorative dentistry because of their favorable mechanical and esthetic properties. This in vitro study evaluated the effect of acidic exposure on surface properties and *Streptococcus mutans* biofilm formation on three CAD/CAM resin composites: a short-fiber-reinforced composite (SFRC), a woven glass fiber-reinforced composite (TRINIA; TR), and a resin nanoceramic-reinforced composite (Lava Ultimate; LU). Twenty-four specimens from each material were prepared and assessed for surface roughness and wettability using an optical profilometer and contact angle goniometer before and after 24 h of storage in neutral (pH 7) or acidic (pH 4) buffer solutions (n = 12 per group). Surface morphology was further examined using scanning electron microscopy (SEM). Biofilm formation was quantified using *S. mutans* colony-forming unit (CFU) counts, followed by confocal laser scanning microscopy (CLSM) for biofilm architecture and viability. At baseline, TR exhibited the highest roughness values (0.744 to 0.785 µm, *p* < 0.001), whereas no significant differences in contact angle were observed among materials. Acidic exposure significantly increased surface roughness and reduced contact angle values in all examined materials (*p* < 0.001). Compared to storage in neutral conditions, bacterial adhesion was significantly reduced in TR and LU stored in acid (*p* < 0.001), but SFRC showed no significant change (*p* = 0.079). Among specimens exposed to acidic conditions, TR exhibited the highest bacterial adhesion (3.24 log_10_ CFU/mm^2^) relative to SFRC and LU (*p* < 0.001). CLSM analysis demonstrated denser biofilm accumulation on TR and LU compared with SFRC. These findings demonstrate that acidic exposure significantly affects the surface properties and bacterial adhesion behavior of CAD/CAM resin composites in a material-dependent manner. SFRC showed no statistically significant difference in *S. mutans* CFU/mm^2^ between neutral and acidic storage conditions despite measurable surface changes, highlighting its potential suitability for clinical applications associated with acidic oral environments.

## 1. Introduction

Computer-aided design and computer-aided manufacturing (CAD/CAM) technology has significantly improved restorative dentistry by enabling highly precise and efficient fabrication of indirect restorations. Although ceramics and metal-based materials have long been used in CAD/CAM workflows, the introduction of resin-based CAD/CAM materials has offered advantages such as easier machinability, reduced brittleness, and favorable esthetic and cost profiles [1,2,3,4]. Despite these improvements, the long-term performance of CAD/CAM resin composites remains a concern due to their susceptibility to fracture and surface degradation [5,6]. To address these limitations, advances in filler- and fiber-reinforcement technologies have been pursued to improve the mechanical properties of resin composites. Among commercially available CAD/CAM resin composites, Lava™ Ultimate (LU), a resin composite comprising nanoceramic particles (80% wt. of silica and zirconia) embedded in a resin matrix, was developed to offer resilience and wear resistance for indirect restorations [3,7]. LU is promoted for its high polishability, modulus of elasticity similar to that of natural dentin, and excellent esthetics [3,5]. However, LU’s long-term clinical performance may be affected by susceptibility to acid-induced surface degradation [8,9]. Other materials incorporated long fibers such as TRINIA (TR), which is a CAD/CAM composite reinforced by bidirectional glass fibers within an epoxy resin matrix, providing enhanced strength along the direction of fiber alignment [8,10,11]. However, TR exhibits anisotropic behavior, making it directionally dependent and less ideal for restorations in complex anatomical regions [11]. To overcome this limitation, short-fiber-reinforced composites (SFRCs) have been developed using randomly oriented, discontinuous glass fibers, which offer isotropic reinforcement and improved performance under multidirectional forces [12,13]. Based on the advancement of filler- and fiber-reinforcing technologies, an experimental short-fiber-reinforced CAD/CAM composite (SFRC) integrating multidirectional short glass fibers (25% wt.) and barium glass filler particles (52% wt.) within a polymer matrix was developed. The SFRC has shown promising preliminary results in terms of fracture resistance, surface integrity, and bonding strength [7,14,15]. However, this CAD/CAM material is still not commercially available. As such, limited data exists regarding its chemical stability and biological behavior under simulated oral conditions, including pH changes and microbial exposure. In the oral environment, restorative materials are continuously exposed to fluctuating pH levels caused by dietary acids, microbial metabolism, and gastroesophageal reflux. These acidic challenges can alter the physical characteristics of resin composites, increasing surface roughness, changing wettability, and weakening the filler–resin interface [9,16,17]. Such degradation not only impacts the mechanical function of the restoration but may also influence its interaction with oral bacteria. One major concern is the formation of biofilms on degraded surfaces. Biofilms are structured microbial communities embedded in a self-produced extracellular polymeric matrix that adheres to both natural and artificial surfaces [18]. This matrix protects the bacteria from environmental stress, enhances resistance to antimicrobials, and supports persistent colonization. In the oral cavity, biofilms form rapidly and are a key factor in the development of secondary caries and periodontal disease. Their formation is significantly influenced by material surface properties, particularly roughness, surface energy, and hydrophilicity, which may be altered following exposure to acids [19,20,21]. Among oral bacteria, *Streptococcus mutans* is a major contributor to cariogenic biofilms and plays a central role in the pathogenesis of dental caries. It adheres preferentially to roughened or hydrophilic surfaces and produces extracellular polysaccharides that facilitate bacterial aggregation and acid production [18,19]. Although biofilm behavior on conventional and CAD/CAM resin composites has been previously investigated with regard to different polymeric content [20,22,23], the response of emerging fiber-reinforced CAD/CAM materials, particularly experimental SFRC CAD materials, to acidic degradation and bacterial colonization remains largely unexplored. Therefore, this study aims to characterize the surface properties of three CAD/CAM resin composites reinforced with either fillers or fibers, and to assess the development of bacterial biofilms following storage in neutral and acidic buffer solutions for 24 h. Unlike TRINIA, which relies on directional woven fiber networks, and Lava Ultimate, which depends on a nanoceramic filler–resin interface, SFRC’s hybridized isotropic architecture may confer greater resistance to acid-induced degradation due to its isotropic reinforcement and more uniform stress distribution. We therefore hypothesized that SFRC would demonstrate distinct surface behavior under acidic challenge and a different pattern of *S. mutans* adhesion compared with TR and LU. The null hypotheses tested were: (i) there are no differences in surface roughness, contact angle, or bacterial adherence among the three CAD/CAM reinforced resin composites; and (ii) surface characterization and bacterial adhesion to these materials are not affected by storage in acidic buffer solution.

## 2. Materials and Methods

### 2.1. Study Design

**Figure 1:**
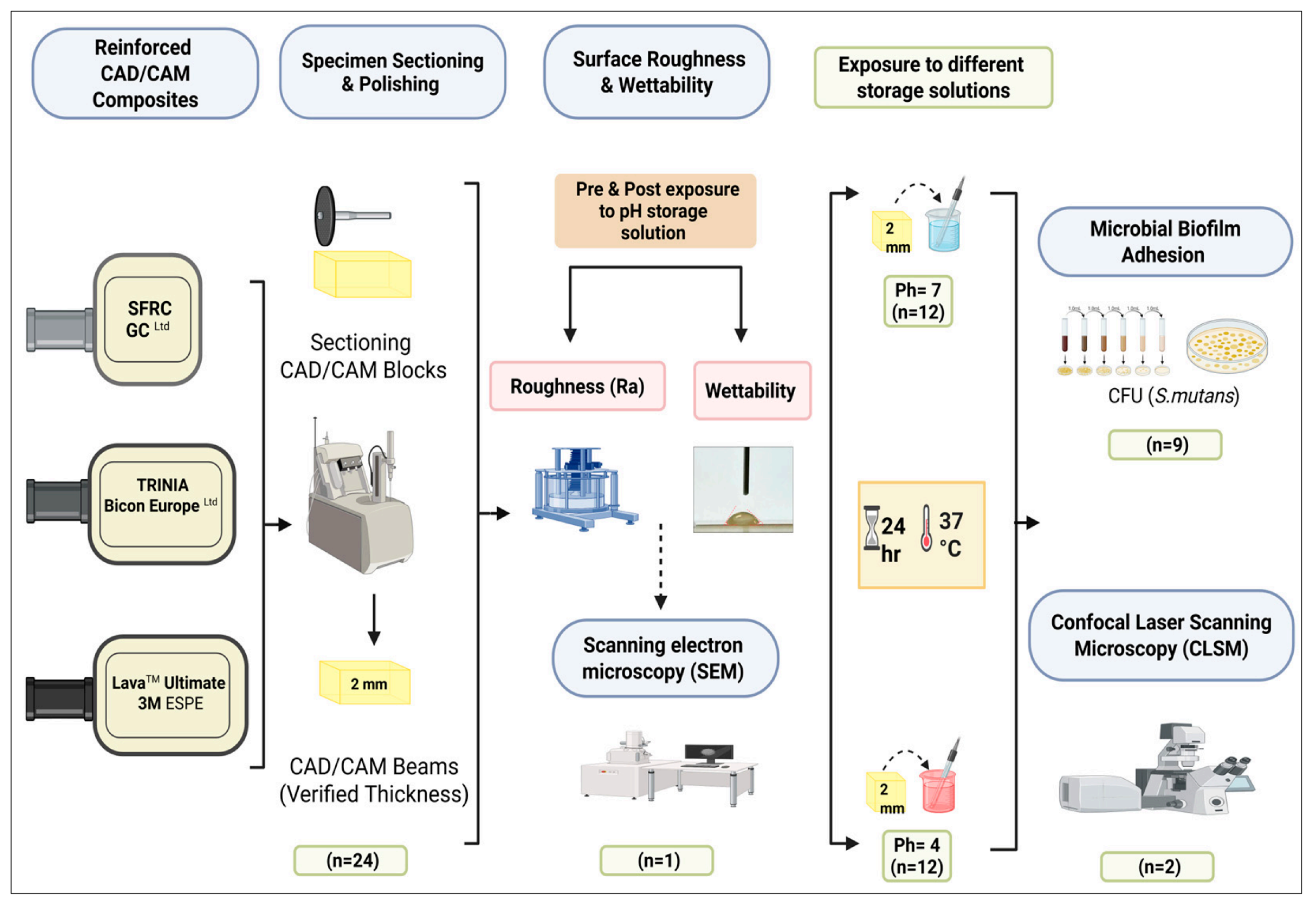
A graphical representation of the study flowchart.

The sample size was determined based on previous studies [17,20]. A total of 72 specimens were prepared from three CAD/CAM reinforced resin composites, (Table 1): an experimental fiber-reinforced composite containing barium glass fillers and short glass fibers (SFRC), a woven glass fiber-reinforced composite (TRINIA), and a nanoceramic-reinforced resin composite (Lava Ultimate). Specimens from each material were divided into two groups and stored in either neutral (pH 7) or acidic (pH 4) buffer solutions, both supplied by Scientific Laboratory Supplies (Hessle, UK). Surface roughness and wettability measurements were performed before and after exposure to the pH storage solutions (n = 24). All specimens were stored in their assigned media for 24 h at 37 °C, and then one specimen from each material was analyzed using scanning electron microscopy (SEM). The bacterial adhesion of *S. mutans* was determined by counting colony-forming units (CFU) for nine specimens, and two specimens were submitted for confocal laser scanning microscopy (CLSM) to further evaluate the effect of acidity on the quality of the adhered biofilm. All specimens were labeled with a code and serial number to ensure consistency and accuracy. The study flowchart is illustrated in Figure 1.

### 2.2. Specimen Preparation

Specimens from each material were sectioned into beams (n = 24) using a diamond disk with a low-speed precision cutting machine under constant water cooling (IsoMet 1000 Precision saw, Buhler, Lake Bluff, IL, USA). The specimens were polished manually using SiC papers of grit sizes P600 and P800 to smooth and round the edges. Specimens were placed in a holding mold and polished manually under dry conditions using regular strokes in one direction for 30 s per SiC paper (P600 and P800) by a single operator. Polishing was performed without applied water cooling, and the holding mold ensured consistent specimen positioning throughout the procedure. Specimen dimensions were measured using a digital caliper for beams (8 mm × 4 mm × 2 mm) and then ultrasonically cleaned for 5 min in distilled water.

### 2.3. Surface Roughness

Surface roughness of the specimens (n = 24) was evaluated. The same specimens were evaluated before and after exposure to pH storage solutions using an optical profilometer (Talysurf CLI 1000, Taylor Hobson Precision, Leicester, UK). High-resolution scans were performed on two distinct areas per specimen, arranged in a grid pattern. Each location was measured at three intervals, and the mean value was calculated for each site. Measurements were conducted using vertical-scan interferometry with a 0.9 mm × 0.7 mm scan area at 1× speed and 5× magnification. Surface roughness was quantified as the arithmetic mean roughness (Ra, in µm), following the definition provided by ISO 25178/2017 [24].

### 2.4. Wettability (Contact Angle Measurements)

Contact angle measurements were obtained before and after exposure to pH storage solutions using the sessile drop method via a calibrated Goniometer (Ramé-Hart 100-FO, Netcong, NJ, USA). For each specimen (n = 24), a 2.0 µL droplet of distilled water was placed on the surface, and the right and left contact angles were recorded after 20 s using DROPimage software (Version 2.5.01), in accordance with previously established protocols [20,25]. Measurements were conducted under standard laboratory conditions; temperature and humidity were not formally controlled but remained within 23 °C and 40–60% humidity typical of an air-conditioned laboratory environment. Each specimen was measured twice, and the mean contact angle (±SD) was calculated and reported in degrees.

### 2.5. Scanning Electron Microscopy (SEM)

One representative specimen from each material and storage condition (baseline, neutral, and acidic) was mounted on a metal stub using double-sided carbon adhesive tape and ultrasonically cleaned in distilled water for 15 min to remove debris, followed by air drying for 60 s. Specimens were sputter-coated with a thin gold film (~10 nm; Quorum Q150R, Quorum Technologies, Lewes, UK, 1 min) and examined using a JEOL JSM-6610LV scanning electron microscope (JEOL Co., Tokyo, Japan) in backscattered electron mode at an accelerating voltage of 10 kV, a working distance of 10 mm, and a magnification of 200×.

### 2.6. Microbiological Procedure

*S. mutans* strain ATCC 25,175 (American Type Culture Collection, Manassas, VA, USA) was obtained from the microbiology laboratory’s culture collection. The strain was cultured on brain heart infusion (BHI) agar (Difco, Detroit, MI, USA) and incubated at 37 °C for 24 h in a 10% CO_2_ atmosphere. After incubation, bacterial colonies were harvested and suspended in phosphate-buffered saline (PBS; Sigma-Aldrich, Darmstadt, Germany). The bacterial suspension was then standardized to a 0.5 McFarland turbidity standard, corresponding to an approximate concentration of 1.5 × 10^8^ CFU/mL.

#### 2.6.1. In Vitro Biofilm Adhesion Assay

Nine sterilized beam specimens (autoclaved at 121 °C for 15 min at 15 psi) from each study group (neutral pH 7 and acidic pH 4 buffer solutions, Scientific Laboratory Supplies, Hessle, UK) were transferred into sterile Petri dishes containing 5 mL of sterile artificial saliva (Sigma-Aldrich, Germany), composed of 0.126 g/L NaCl, 0.964 g/L KCl, 0.189 g/L KSCN, 0.655 g/L KH_2_PO_4_, and 0.200 g/L urea, and incubated at 37 °C in a 10% CO_2_ atmosphere for 1 h to allow pellicle formation. Each specimen was then placed into an individual well of a sterile 24-well tissue culture plate, and 2 mL of a standardized *S. mutans* suspension (0.5 McFarland; ~1.5 × 10^8^ cells/mL) was added. The plates were incubated for 24 h at 37 °C in a CO_2_ incubator to promote bacterial adhesion and initial biofilm development. After incubation, specimens were gently rinsed twice with sterile PBS to remove non-adherent bacteria. Adherent biofilms were dislodged by vortexing and sonicating each specimen in 1.5 mL of sterile PBS for 30 s. Adherent biofilms were dislodged by vertexing using Branson Bransonic M1800 ultrasonic cleaner (Emerson Electric Co., St. Louis, MO, USA) operating at 40 kHz. The resulting bacterial suspensions were serially diluted, and 0.1 mL aliquots were plated on BHI agar and incubated for 48 h at 37 °C in a 10% CO_2_ atmosphere. Colony-forming units (CFU) of *S. mutans* were counted using a darkfield colony counter (Reichert Quebec^®^, Cambridge Instruments, Buffalo, NY, USA) and expressed as CFU/mm^2^, normalized to the exposed flat surface area of each beam specimen (32 mm^2^), applied consistently across all experimental groups.

#### 2.6.2. Confocal Laser Scanning Microscopy (CLSM)

CLSM images were used as a representative qualitative imaging technique to assess biofilm distribution and viability; two beam specimens were randomly selected from each material in each study group (neutral and acidic conditions). The selected specimens were gently retrieved from the wells, rinsed twice with sterile PBS to remove non-adherent cells, and stained using the LIVE/DEAD™ BacLight™ Bacterial Viability Kit (Molecular Probes, Eugene, OR, USA). The stained specimens were mounted and imaged using a Nikon C2 confocal laser scanning microscope (Nikon Instruments Inc., Melville, NY, USA) equipped with a 10×/0.49 NA air immersion objective. Four regions (one from each corner) of each specimen were scanned. Live bacterial cells were visualized using excitation at 488 nm, with emission collected through a 520–540 nm bandpass filter (green channel). Dead cells were detected using excitation at 568 nm, with emission captured through a 600–630 nm bandpass filter (red channel). CLSM images were used to qualitatively assess biofilm distribution and viability.

### 2.7. Statistical Analysis

Statistical analyses were performed using Python 3.12 (SciPy 1.x, Statsmodels, and scikit-posthocs). Normality was assessed using the Shapiro–Wilk test and homogeneity of variance using Levene’s test. A significance level of α = 0.05 was applied throughout, and Bonferroni correction was applied where multiple comparisons were performed. For surface roughness (Ra) and contact angle, nonparametric tests were used as most datasets violated the assumptions of normality and/or homogeneity of variance. Between-material comparisons at the same time point were performed using the Kruskal–Wallis test, followed by Dunn’s post hoc analysis with Bonferroni correction, except for the acidic post-storage roughness data, which met the assumptions of homogeneity of variance and was therefore analyzed using Welch ANOVA with Games–Howell post hoc. Within-material pre-to-post comparisons were performed using the Wilcoxon signed-rank test or paired *t*-test depending on data distribution. Between-condition comparisons (neutral vs. acidic, post-storage) were performed using Mann–Whitney U tests or independent *t*-tests as appropriate. For bacterial adhesion, *S. mutans* CFU/mm^2^ values were log_10_-transformed prior to analysis to stabilize variance and normalize the distribution. The transformed data met the assumptions of normality and homogeneity of variance; therefore, a two-way ANOVA was used to evaluate the main effects of material type and storage condition and their interaction, followed by Games–Howell post hoc comparisons. A post hoc power analysis was conducted for the primary outcome variable (log_10_-transformed *S. mutans* CFU/mm^2^) using the obtained effect size from the one-way ANOVA comparing the three materials post-storage (η^2^ = 0.656, Cohen’s f = 1.380), with α = 0.05 and n = 9 specimens per group. The achieved statistical power was >0.999, confirming that the sample size was more than adequate to detect the observed between-group differences.

## 3. Results

### 3.1. Surface Roughness (Ra)

Baseline surface roughness differed significantly among the three CAD/CAM composites (*p* < 0.001), ranging from 0.198 µm to 0.785 µm, with TR exhibiting the highest Ra values, followed by LU and SFRC. This pattern remained unchanged after storage under both neutral and acidic conditions (Table 2). Acidic storage significantly increased Ra in all materials, with the greatest increase in TR, followed by LU and SFRC (*p* < 0.001). Neutral storage produced no significant change in SFRC or LU and only a small but statistically significant increase in TR (*p* = 0.008).

### 3.2. Wettability (Contact Angle)

No significant differences in baseline contact angle were observed among the three materials (*p* = 0.191), with values ranging from 63.65 to 67.57. No inter-material differences were detected after storage in either pH storage solution exposure (Table 3). Acidic exposure significantly reduced the contact angle in SFRC, TR, and LU (all *p* < 0.001), indicating increased surface hydrophilicity, whereas neutral storage produced only small but statistically significant reductions. The contact angle reduction was significantly greater after exposure to the acidic solution than after exposure to the neutral solution for all materials (*p* < 0.001).

### 3.3. Surface Analysis by SEM

**Figure 2:**
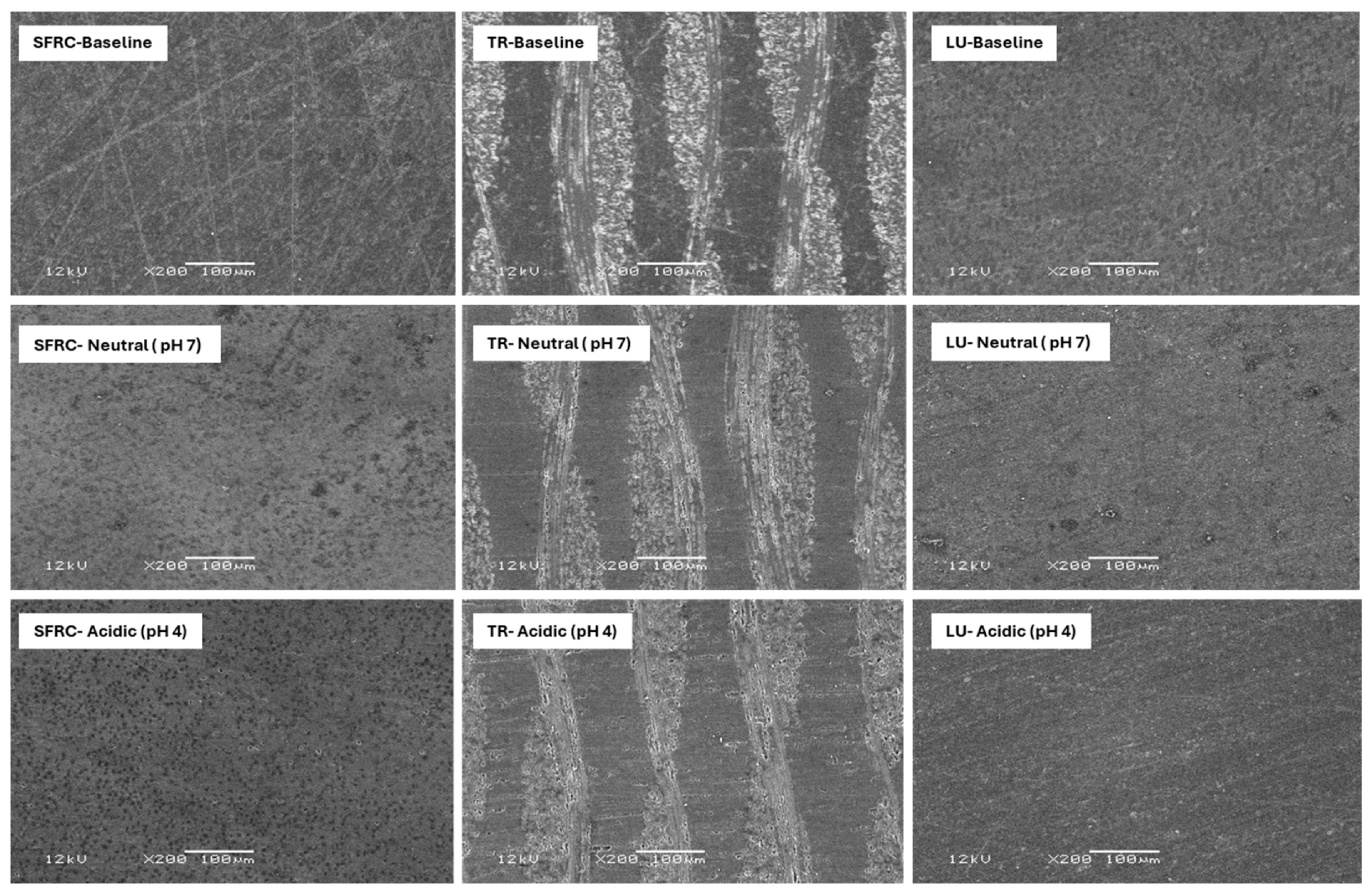
Representative SEM images (×200 magnification) of SFRC, TR, and LU specimens after immersion in neutral and acidic buffer (pH 4, pH 7).

SEM was used as a qualitative complement to the profilometry data, providing visual characterization of acid-induced morphological changes at the material surface. Microstructural differences among the materials were visualized in SEM images of the reinforced resin composite surfaces before and after storage in neutral and acidic media (Figure 2). Both SFRC and TR are reinforced with glass fibers; however, SFRC contains short, randomly distributed fibers interspersed with filler particles, while TR features woven, linear fibers without filler particles. In contrast, LU is reinforced with ceramic nanofiller particles, resulting in a comparatively smoother surface. Immersion in buffer solutions, regardless of pH, altered the surface characteristics of all three materials relative to the baseline dry condition, with each material exhibiting varying degrees of visible exposure of filler particles and localized matrix dissolution.

### 3.4. Colony-Forming Units

**Figure 3:**
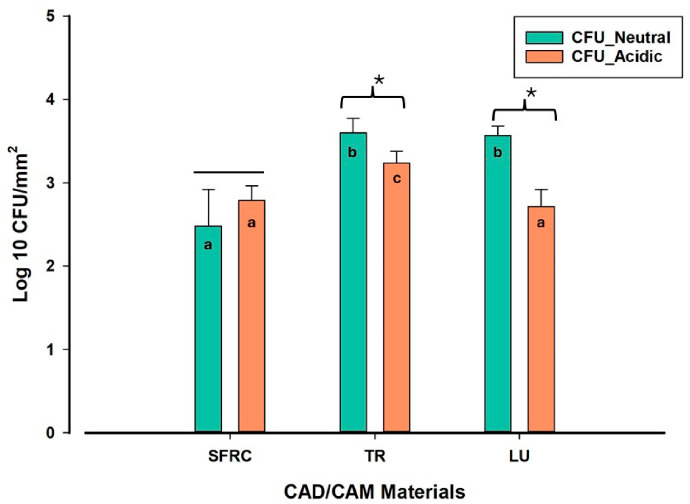
Mean (SD) *S. mutans* bacterial counts (log_10_ CFU/mm^2^) on SFRC, TR, and LU specimens after 24 h of biofilm incubation following neutral and acidic storage exposure. An asterisk (*) indicates a statistically significant difference compared with the corresponding neutral condition within the same material (*p* < 0.05). Different lowercase letters indicate statistically significant differences among materials within the same storage condition (Games–Howell post hoc test, *p* < 0.05).

Two-way ANOVA revealed a statistically significant interaction between material type and storage condition on *S. mutans* adhesion (*p* < 0.001). Significant main effects of both material and storage condition were also observed (both *p* < 0.001) (Table 4), while group means are summarized in Table 5. Under neutral conditions, no significant difference in CFU counts was observed between TR and LU (*p* = 0.892), whereas SFRC exhibited significantly lower bacterial adhesion than either material (*p* < 0.001). Following acidic storage, adhesion to SFRC was not significantly affected compared with neutral conditions (*p* = 0.079). In contrast, significant reductions in bacterial adhesion were observed in TR and LU after acidic exposure (both *p* < 0.001). Consequently, TR stored under acidic conditions demonstrated significantly greater bacterial adhesion than both SFRC and LU (*p* < 0.001), while no significant difference was detected between SFRC and LU (*p* = 0.696) (Figure 3).

### 3.5. Surface Analysis CLSM

**Figure 4:**
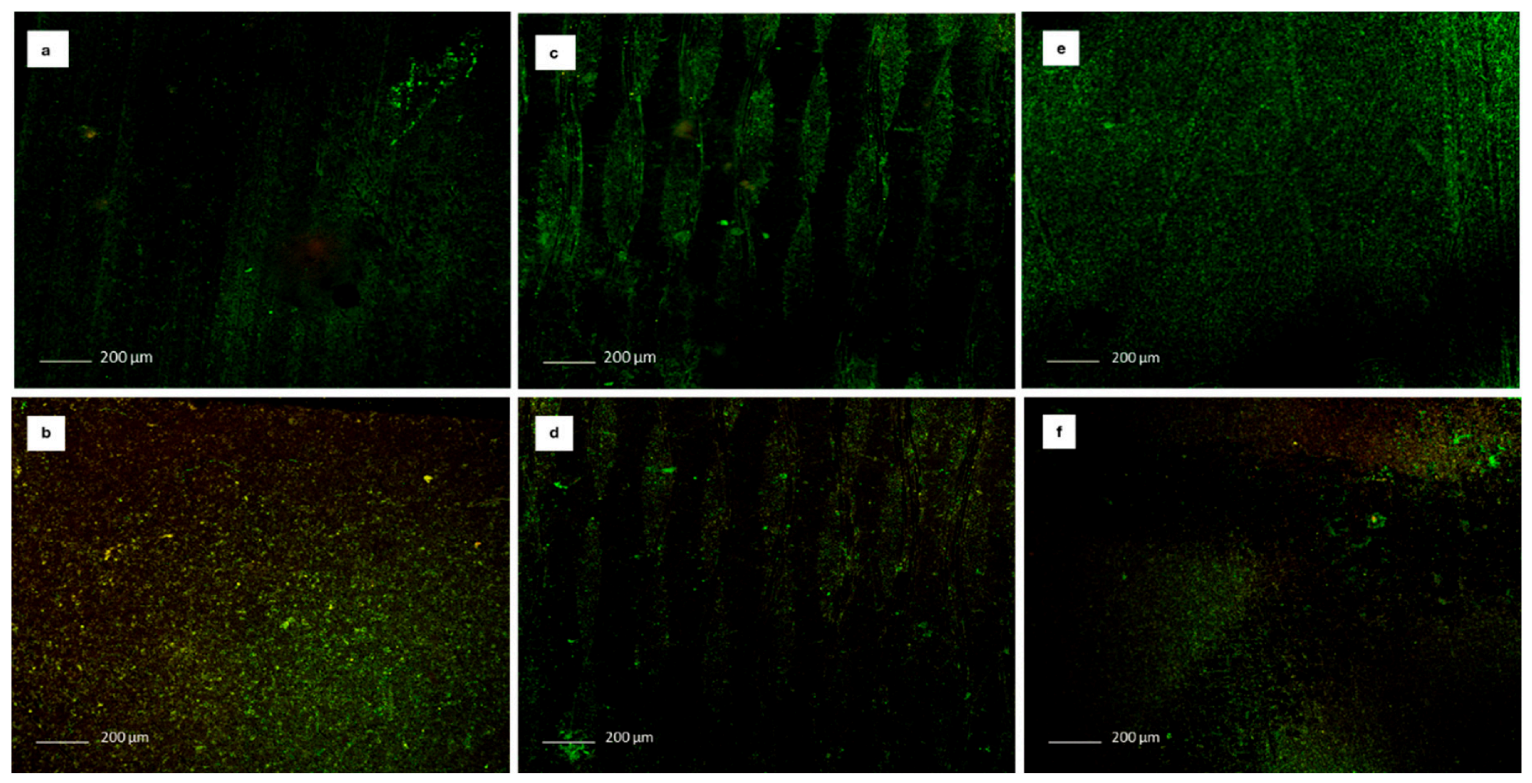
Representative CLSM images of *S. mutans* on the surfaces of SFRC (**a**,**b**), TR (**c**,**d**), and LU (**e**,**f**) specimen surfaces after 24 h of immersion under neutral (pH 7; (**a**,**c**,**e**)) and acidic (pH 4; (**b**,**d**,**f**)) storage conditions.

CLSM imaging revealed biofilm patterns that corresponded to these microstructural differences (Figure 4). Consistent with the CFU results, TR and LU demonstrated greater bacterial adherence than SFRC under neutral conditions. Following storage in acidic solutions, CFU counts were reduced for TR and LU, whereas SFRC exhibited a slight increase in bacterial colonization.

## 4. Discussion

This study evaluated the effect of short-term acidic storage on surface properties and *S. mutans* adhesion to three CAD/CAM reinforced resin composites with distinct reinforcement strategies: a short-fiber-reinforced composite (SFRC), a woven glass fiber-reinforced composite (TRINIA; TR), and a nanoceramic-reinforced resin composite (Lava Ultimate; LU). The findings demonstrated material-dependent differences in surface roughness, wettability, and bacterial adhesion, as well as variable responses to acidic exposure among the tested materials. Overall, the null hypotheses were partially rejected. Our previous study demonstrated that material composition and surface characteristics significantly influence *S. mutans* biofilm formation on high-performance polymeric CAD/CAM composites under neutral conditions [20]. However, the combined effect of acidic exposure on the surface properties and bacterial adhesion of esthetically reinforced CAD/CAM composites remains underexplored. In the present study, acidic storage (pH 4, 24 h) significantly altered surface roughness and wettability in all tested materials, with corresponding material-dependent differences in *S. mutans* adhesion. These findings may be clinically relevant in acidic oral environments associated with frequent sugar consumption, gastric reflux, or poor oral hygiene.

Lava Ultimate (LU), a well-established nanoceramic CAD/CAM composite, was used as a benchmark material in this study. At baseline, LU exhibited intermediate surface roughness and contact angle values compared with TR and SFRC. All specimens were polished to P800 grit to standardize surface finishing and minimize initial surface variability among the tested materials prior to biofilm assessment. Despite its relatively smooth surface characteristics, LU demonstrated high *S. mutans* adhesion under neutral conditions, indicating that low roughness and wettability alone may not fully prevent early biofilm formation. These findings are consistent with those of Park et al. (2012), who reported that surface free energy and material composition play important roles in bacterial adhesion, in addition to surface roughness and hydrophilicity [19]. TRINIA (TR), composed of woven bidirectional glass fibers embedded within an epoxy matrix, exhibited the highest surface roughness values under both neutral and acidic conditions. Such surface characteristics have previously been associated with increased microbial retention [20,26]. Consistent with these findings, TR demonstrated the highest *S. mutans* adhesion under neutral storage conditions [20,21].

The woven fiber structure of TR may create surface irregularities and microgrooves that facilitate bacterial retention and accumulation. A similar trend was reported by Suzaki et al. (2020), who highlighted the roughness and anisotropic surface characteristics of fiber-reinforced composites as potential limitations in microbial environments despite their favorable mechanical properties [11]. In contrast, SFRC exhibited the lowest *S. mutans* adhesion despite showing intermediate roughness and wettability values. Its structure, composed of randomly oriented short glass fibers and barium glass fillers embedded within a polymer matrix, may contribute to a more homogeneous surface morphology and isotropic behavior [10,12,13]. Interestingly, although acidic exposure significantly increased surface roughness in SFRC, bacterial adhesion remained relatively stable, suggesting that factors beyond roughness alone may influence microbial colonization on this material. This observation is supported by Lassila et al. (2023), who reported that the same SFRC formulation maintained favorable surface integrity and bonding performance following thermal aging and mechanical fatigue [15].

Acidic storage significantly affected bacterial adhesion in LU and TR, resulting in reduced CFU counts, whereas *S. mutans* adhesion to SFRC remained statistically unchanged. Post-storage surface analysis demonstrated that acidic exposure increased surface roughness and reduced contact angles across all materials, indicating greater surface hydrophilicity following acid immersion. Despite these surface alterations, the bacterial response varied across materials. No formal correlation analysis was performed between surface parameters and bacterial adhesion counts; therefore, the observed patterns should be interpreted as descriptive rather than causally linked. *S. mutans* adhesion is mediated through specific adhesin–receptor interactions that are sensitive to surface chemistry rather than surface topography alone, meaning that acid-induced alterations to surface chemistry may override the pro-adhesive effect of increased roughness. The greater chemical susceptibility of LU and TR to acid-induced degradation may therefore paradoxically render their surfaces less conducive to bacterial colonization, as discussed in detail below.

LU has previously been reported to undergo hydrolytic degradation at the resin–filler interface following exposure to acidic environments, resulting in filler debonding and increased porosity [9]. Such surface alterations may influence bacterial–substrate interactions and biofilm retention. Similarly, TR exhibited the greatest increase in surface roughness after acidic storage, likely due to the susceptibility of the woven fiber structure and epoxy matrix to acid-induced degradation. Despite increased roughness, TR exhibited reduced *S. mutans* adhesion after acidic exposure. Comparable observations have been reported in glass fiber-reinforced composites exposed to acidic beverages, where matrix degradation altered surface integrity and bacterial compatibility [5].

SFRC demonstrated relatively stable *S. mutans* adhesion under both storage conditions, despite a significant increase in surface roughness after acidic exposure. Unlike LU and TR, which rely predominantly on ceramic fillers or directional fiber networks, SFRC contains a hybridized filler–fiber structure that may contribute to more stable bacterial interactions under acidic conditions. These findings suggest that factors beyond surface roughness alone may influence microbial colonization on reinforced CAD/CAM composites. Whether such properties translate into clinical advantages for restorations in posterior regions or for high-caries-risk patients warrants further investigation. Similar observations were highlighted by Papathanasiou et al. (2023), who emphasized the importance of acid-resistant CAD/CAM materials for long-term clinical performance in acidic oral environments [7].

CLSM and SEM observations supported the CFU findings. TR and LU exhibited denser and more complex biofilm structures under neutral conditions, whereas biofilm organization appeared visibly disrupted following acidic exposure. In contrast, SFRC demonstrated comparatively sparse and loosely organized biofilm structures under both storage conditions. CLSM imaging was performed on two representative specimens per group to provide qualitative visualization of biofilm distribution and viability patterns, and the observations were consistent with the quantitative CFU data. It should be noted, however, that SEM imaging was performed at 200× magnification, limiting the resolution of submicron surface features such as filler debonding or matrix degradation, and CLSM was conducted on two representative specimens per group without quantitative biofilm parameters; both, therefore, serve as qualitative complements to the profilometry and CFU data rather than independent quantitative outcomes. These findings support previous microscopy-based studies indicating that biofilm architecture is influenced by surface topography, surface energy, and material microstructure [18].

Interestingly, no direct association was found between surface properties and bacterial adhesion, despite significant acid-induced changes in surface roughness and wettability. This finding highlights the multifactorial nature of biofilm formation. In addition to surface characteristics, bacterial colonization may be influenced by material composition, pellicle formation, protein adsorption, and microbial interactions [27,28,29].

Although CAD/CAM materials are polymerized under industrial conditions and generally achieve higher degrees of conversion than chairside composites, residual unpolymerized monomers are not fully eliminated [22]. Under acidic conditions, these monomers may leach into the surrounding environment. Other degradation products may also be released through hydrolysis of the organic matrix. This process has been well described for Bis-GMA- and TEGDMA-containing methacrylate systems [22,30,31]. At the filler–matrix interface, low pH can promote silane hydrolysis. This may lead to filler particle detachment, matrix plasticization, and material-specific patterns of surface degradation [22,30].

TRINIA has an epoxy-impregnated woven glass-fiber architecture. This differs fundamentally from the silanized-particle methacrylate networks of SFRC and Lava Ultimate. Therefore, TRINIA may generate a chemically distinct leachate profile after acid exposure. Since composite formulation strongly influences the nature and extent of chemical degradation [30,31], differences in leachate profiles among the three materials may have affected surface–bacteria interactions. These effects may not be fully captured by roughness and contact angle measurements alone.

The chemical identity of the pH 4 buffer components should also be considered. Buffer-derived ions or molecules may have remained adsorbed on the more porous surfaces of TR and LU after the post-storage washing procedure. These residues could have interfered with subsequent pellicle formation or early bacterial adhesion.

Acidic exposure significantly reduced bacterial adhesion in LU and TR. In contrast, SFRC maintained relatively stable *S. mutans* adhesion under both storage conditions. The inclusion of LU as a benchmark material showed that emerging reinforced composites may display favorable biological behavior under acidic stress. These preliminary in vitro findings suggest that biological performance under acidic conditions may be relevant when selecting materials, alongside mechanical and esthetic properties. However, clinical conclusions cannot be drawn from a single-species 24 h model. Further studies using multi-species biofilm models and longer aging protocols are needed before clinical recommendations can be made.

Within the limitations of this study, the three materials exhibited markedly different baseline surface roughness values before acidic storage, reflecting their distinct microstructural architectures. Therefore, direct comparisons of bacterial adhesion between materials should be interpreted with caution. The observed differences may be partly attributable to pre-existing surface characteristics rather than to acidic exposure alone. Future studies using ANCOVA, with baseline roughness as a covariate, may help clarify this contribution.

In addition, SFRC showed relatively stable bacterial adhesion after acidic exposure, despite measurable surface alterations. Further studies should include surface chemical characterization, such as XPS or ATR-FTIR, before and after acidic exposure. Direct measurement of monomer and ion release using high-performance liquid chromatography (HPLC) is also recommended. Monitoring the pH of the culture medium would provide additional insight. These approaches may help determine whether leachate chemistry or surface-adsorbed buffer residues influence *S. mutans* adhesion independently of macroscopic surface properties.

Future in vivo studies, multi-species biofilm models, and longer aging protocols are also needed. These would help validate the present findings and improve their clinical relevance.

## 5. Conclusions

This study demonstrated that *S. mutans* adhesion to CAD/CAM resin composites is material-dependent and influenced by acidic exposure. Acidic storage significantly affected surface roughness and wettability across all tested materials; however, the bacterial response varied with material composition and reinforcement strategy. The short-fiber-reinforced composite (SFRC) exhibited the lowest bacterial adhesion under both neutral and acidic conditions and maintained relatively stable biofilm formation despite measurable surface alterations following acidic exposure. In contrast, TRINIA (TR) and Lava Ultimate (LU) showed significant reductions in bacterial adhesion after acidic storage. These findings suggest that reinforced CAD/CAM composites exhibit distinct biological responses under acidic conditions and highlight the potential of SFRC as a promising material for clinical situations associated with acidic oral environments or increased biofilm challenge. Further in vivo studies and long-term aging investigations are recommended to confirm these findings and evaluate their clinical relevance.

## Figures and Tables

**Table 1:** Reinforced CAD/CAM resin composites and their manufacturer information.

Code	Materials	Product Name	Composition	Manufacturer
**SFRC**	Hybrid FRC	Experimental short-fiber-reinforced composite CAD material	52 wt.% barium glass 25 wt.% glass fibers 23 wt.% UDMA, TEGDMA, short (200–300 µm length), 7 µm diameter, discontinuous, 3D oriented glass fibers.	Stick Tech Ltd., GC Europa AG Company, Turku, Finland
**TR**	Woven FRC	TRINIA	55–60 wt.% glass fiber 40–45 wt.% epoxy resin, woven glass fibers.	Bicon Europe, Ltd., Leuven, Belgium
**LU**	Resin nanoceramic composite	Lava Ultimate	86 wt.% open porous feldspathic ceramic structure networks. 14 wt.% of cross-linked polymers (UDMA, TEGDMA)	3M^TM^ESPE^TM^, St. Paul, MN, USA
**pH4**	pH 4	Acidic buffer solution (blue)	Water; 1,2-Benzenedicarboxylic acid, monopotassium salt; formaldehyde; methyl alcohol; fluorescein, 2′,4′,5′,7′-tetraiodo, disodium salt.	Scientific Laboratory Supplies (SLS)
**pH7**	pH 7	Neutral buffer solution (yellow)	Water; dihydrogen potassium phosphate; sodium hydroxide; FD&C yellow No. 5.	

**Table 2:** Means and standard deviations of roughness (Ra) measurements of three CAD/CAM resin composites (SFRC, TR, and LU) before and after storage in neutral and acidic buffer solutions.

	Ra (µm)			
Material	Neutral (pH 7)		Acidic (pH 4)	
	Pre	Post	Pre	Post
**SFRC**	0.198 (0.005) **^a^**	0.197 (0.006) **^a^**	0.210 (0.012) **^a^**	0.241 (0.020) ^a,^*
**TR**	0.785 (0.098) **^c^**	0.802 (0.098) ^c,^*	0.744 (0.083) **^c^**	0.909 (0.175) ^c,^*
**LU**	0.445 (0.130) **^b^**	0.449 (0.133) **^b^**	0.366 (0.041) **^b^**	0.408 (0.057) ^b,^*

Different superscript lowercase letters indicate statistically significant differences among the three materials within the same column (*p* < 0.05). An asterisk (*) indicates a significant change from the corresponding Pre value within the same material and storage condition (paired test, Bonferroni-adjusted *p* < 0.05). Between-material comparisons used Kruskal–Wallis with Dunn’s Bonferroni-adjusted post hoc test, except for Acidic Post, for which Welch ANOVA with the Games–Howell post hoc test was used.

**Table 3:** Means and standard deviations of the water contact angle of the three CAD/CAM resin composites (SFRC, TR, and LU), before and after storage in neutral and acidic buffer solutions.

Material	Neutral (pH 7)		Acidic (pH 4)	
	Pre	Post	Pre	Post
**SFRC**	67.27 (3.42) **^a^**	65.83 (3.23) ^a,^*	67.03 (3.26) **^a^**	52.21 (5.48) ^a,^*
**TR**	67.57 (9.55) **^a^**	65.88 (9.24) ^a,^*	64.97 (9.54) **^a^**	52.29 (8.05) ^a,^*
**LU**	65.86 (5.73) **^a^**	64.81 (5.87) ^a,^*	63.65 (6.44) **^a^**	52.44 (5.88) ^a,^*

Same superscript lowercase letters indicate no statistically significant differences among the three materials within the same column (*p* > 0.05). An asterisk (*) indicates a statistically significant difference from the corresponding Pre value within the same material and storage condition (paired test, Bonferroni-adjusted *p* < 0.05).

**Table 4:** Two-way ANOVA analysis of the effects of material type and storage condition on *S. mutans* adhesion to CAD/CAM resin composites.

Source	Type III Sum of Squares	df	Mean Square	F	*p*-Value
**Corrected Model**	9.958	5	1.992	36.282	0.000
**Intercept**	507.142	1	507.142	9239.332	0.000
**Storage Condition**	1.239	1	1.239	22.566	0.000
**Materials**	5.676	2	2.838	51.701	0.000
**Storage Condition × Material**	3.043	2	1.522	27.722	0.000
**Error**	2.635	48	0.055		
**Total**	519.734	54			
**Corrected Total**	12.592	53			

Dependent variable: log_10_ CFU/mm^2^. Factors included material type (SFRC, TR, and LU) and storage condition (neutral pH 7 and acidic pH 4). The significant interaction between material and storage condition indicates that the effect of acidic exposure on bacterial adhesion differed among the tested materials. R^2^ = 0.791 (adjusted R^2^ = 0.769).

**Table 5:** Means and standard deviations of *S. mutans* adhesion to the three CAD/CAM resin composites (SFRC, TR, and LU) after 24 h of storage in neutral and acidic buffer solutions.

Storage	SFRC	TR	LU
**Neutral (pH 7)**	2.48 (0.44) **^a^**	3.60 (0.17) **^b^**	3.57 (0.11) **^b^**
**Acidic (pH 4)**	2.79 (0.18) **^a^**	3.24 (0.14) ^b,^*	2.71 (0.21) ^a,^*

Different superscript lowercase letters indicate statistically significant differences among materials within the same storage condition (Games–Howell post hoc test, *p* < 0.05). An asterisk (*) indicates a statistically significant difference compared with the corresponding neutral condition for the same material (*p* < 0.05).

## Data Availability

All data analyzed during this study are included in this published article. Additionally, the complete dataset supporting the conclusions of this article is available from the corresponding author and can be accessed upon reasonable request.

## Abbreviations

The following abbreviations are used in this manuscript:

CAD/CAM	Computer-aided design/computer-aided manufacturing
CFU	Colony-forming unit
CLSM	Confocal laser scanning microscope
